# Neoglycoconjugate of Tetrasaccharide Representing One Repeating Unit of the *Streptococcus pneumoniae* Type 14 Capsular Polysaccharide Induces the Production of Opsonizing IgG1 Antibodies and Possesses the Highest Protective Activity As Compared to Hexa- and Octasaccharide Conjugates

**DOI:** 10.3389/fimmu.2017.00659

**Published:** 2017-06-02

**Authors:** Ekaterina A. Kurbatova, Nelli K. Akhmatova, Elina A. Akhmatova, Nadezhda B. Egorova, Natalya E. Yastrebova, Elena V. Sukhova, Dmitriy V. Yashunsky, Yury E. Tsvetkov, Marina L. Gening, Nikolay E. Nifantiev

**Affiliations:** ^1^Laboratory of Therapeutic Vaccines, Mechnikov Research Institute for Vaccines and Sera, Moscow, Russia; ^2^Laboratory of Glycoconjugate Chemistry, N. D. Zelinsky Institute of Organic Chemistry, Russian Academy of Sciences, Moscow, Russia

**Keywords:** *Streptococcus pneumoniae* type 14, synthetic oligosaccharide, glycoconjugate vaccine protective activity, antibody specificity, opsonophagocytosis, biotinylated oligosaccharide

## Abstract

Identifying protective synthetic oligosaccharide (OS) epitopes of *Streptococcus pneumoniae* capsular polysaccharides (CPs) is an indispensable step in the development of third-generation carbohydrate pneumococcal vaccines. Synthetic tetra-, hexa-, and octasaccharide structurally related to CP of *S. pneumoniae* type 14 were coupled to bovine serum albumin (BSA), adjuvanted with aluminum hydroxide, and tested for their immunogenicity in mice upon intraperitoneal prime-boost immunizations. Injections of the conjugates induced production of opsonizing anti-OS IgG1 antibodies (Abs). Immunization with the tetra- and octasaccharide conjugates stimulated the highest titers of the specific Abs. Further, the tetrasaccharide ligand demonstrated the highest ability to bind OS and CP Abs. Murine immune sera developed against tetra- and octasaccharide conjugates promoted pathogen opsonization to a higher degree than antisera against conjugated hexasaccharide. For the first time, the protective activities of these glycoconjugates were demonstrated in mouse model of generalized pneumococcal infections. The tetrasaccharide conjugate possessed the highest protective activities. Conversely, the octasaccharide conjugate had lower protective activities and the lowest one showed the hexasaccharide conjugate. Sera against all of the glycoconjugates passively protected naive mice from pneumococcal infections. Given that the BSA-tetrasaccharide induced the most abundant yield of specific Abs and the best protective activity, this OS may be regarded as the most promising candidate for the development of conjugated vaccines against *S. pneumoniae* type 14 infections.

## Introduction

*Streptococcus pneumoniae* are Gram-positive bacteria that cause invasive and non-invasive, often lethal, infections in multiple anatomic locations in adults and children (1, 2). Pneumococci capsules are one of the major virulence factors for this class of bacteria (3). Based on the chemical structure of capsular polysaccharides (CPs), more than 90 different serotypes of *S. pneumoniae* have been identified, approximately 20 of which are responsible for 80–90% of all pneumococcal infections (4, 5).

Epidemiologic data have shown that vaccination is an effective way to prevent pneumococcal infection. Studies of unconjugated polysaccharide-based pneumococcal vaccine of the first-generation confirmed its efficacy and safety in adults (6). At the same time, disadvantages of such vaccines have been observed, including inefficiency in children less than 2 years of age and in certain risk groups (7), absence of boosting effects upon revaccination, suggesting insufficient development of immune memory (8).

These disadvantages of polysaccharide vaccines have been overcome in carbohydrate vaccines of the second-generation consisting of CP conjugated to a protein carrier. This results in switching the syntheses of antibodies (Abs) to the carbohydrate component of the conjugate from IgM to IgG, their affinity maturation, formation of immunological memory, and protection of the host from infection by inducing complement-mediated opsonophagocytosis (8–11). The use of pneumococcal conjugate vaccines of the second-generation based on CP of clinically relevant serotypes of *S. pneumoniae* led to a significant reduction in the incidence of pneumococcal infections (5).

However, the use of native CP for production of conjugated vaccines has a number of disadvantages connected with difficulties in bacteria cultivation, isolation, and purification of CP and, in some cases, unsuccessful conjugation of CP to protein carriers (12). A promising direction is the development of carbohydrate pneumococcal vaccines of the third-generation based on synthetic oligosaccharides (OSs) related to the structurally defined regions of CP coupled to protein carriers (13).

To date, the structures of pneumococcal CP of different serotypes have been well described (14). Numerous synthetic OSs that bear structural similarities to CP of serotypes 1–4, 6A/B, 7F, 8, 9A/V, 14, 17F, 18C, 19A/F, 22F, 23F, 27, and 29 have been characterized (15). Several of these OSs have been conjugated to carrier proteins and tested as potential vaccines (13, 16). Advantages of OS-protein conjugate-based vaccines include the absence of bacterial impurities, high serotype specificity of immune responses, and ability of some of them to induce stronger Ab responses compared with traditional conjugated vaccines (16), known and specific engineering of the chemical structures of the synthetic OS allowing for controlled conjugations to carrier proteins, and standardized methods that comply with modern vaccine production requirements. Well-established chemical structures of OS favor to determine the role of specific CP features on the formation of immune responses.

**Figure 1:**
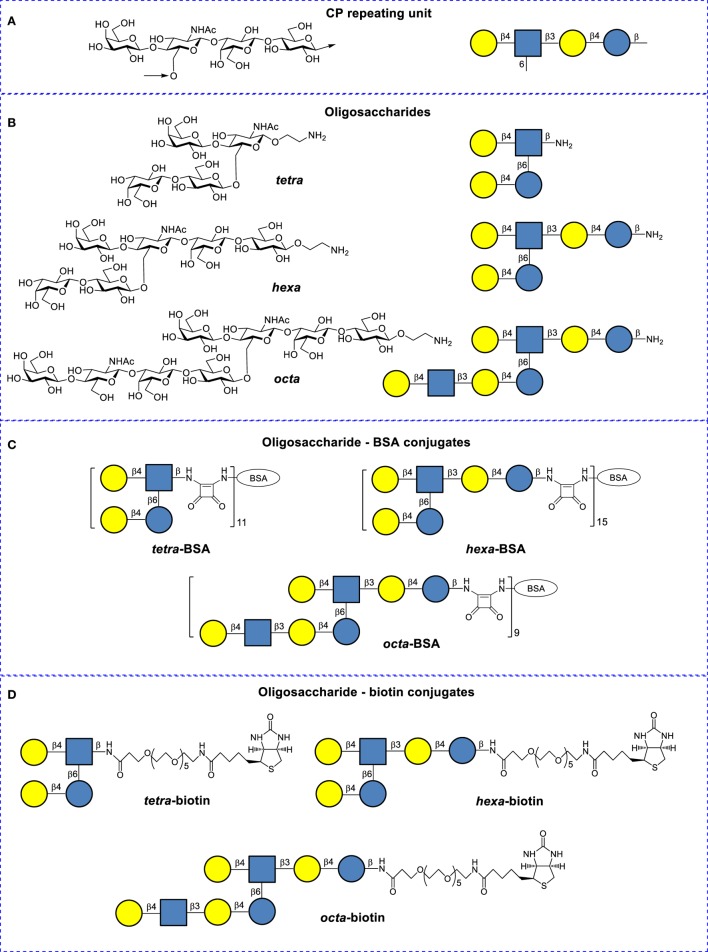
Structures of *Streptococcus pneumoniae* type 14 capsular polysaccharides and their conjugates. **(A)** Repeating unit of the *S. pneumoniae* type 14 CP (structural and symbolic representations). **(B)** Synthetic spacer-armed oligosaccharides (OSs) used to prepare bovine serum albumin (BSA) and biotin conjugates (structural and symbolic representations). **(C)** BSA conjugates of synthetic OSs. **(D)** Biotin conjugates of synthetic OSs.

*Streptococcus pneumoniae* CP type 14 consists of branched tetrasaccharide repeating units (17) (Figure 1). This CP has relatively low immunogenity when compared with other pneumococcal CP serotypes (18). The CP type 14 serotype is very common in the human population (1–3, 19, 20) and frequently infects younger children (14). Previously, the tetrasaccharide ligand (β-d-Gal-(1→4)-β-d-Glc-(1→6)-[β-d-Gal-(1→4)]-β-d-GlcNAc) was described as a good candidate to serve as the *S. pneumoniae* type 14 conjugated vaccine ligand (21). However, these data have not been substantiated with experiments demonstrating protective activity in murine models.

Here, we present for the first time the comparative study of the ability of tetra-bovine serum albumin (BSA), hexa-BSA, and octa-BSA conjugates related to CP of *S. pneumoniae* type 14 to induce anti-CP and anti-OS opsonizing Abs and the evaluation of the efficacy of each neoglycoconjugate in the models of active and passive protection of mice.

## Materials and Methods

### Synthetic OSs and Their Conjugates

Oligosaccharides were conjugated to BSA (Sigma-Aldrich, USA) and biotin (structures and designations are given in Figure 1), as previously described (22, 23). BSA has been frequently used as a protein carrier in engineered immunogenic glycoconjugates and other types of biomolecular systems (24). Matrix laser desorption ionization time-of-flight data demonstrate that the tetra-BSA, hexa-BSA, and octa-BSA conjugates contained on average 11, 15, and 9 OS ligands per protein molecule, corresponding to carbohydrate contents (by weight) of 10, 19, and 16%, respectively (22).

### Synthetic and Bacterial CPs

*Streptococcus pneumoniae* serotype 14 synthetic CP containing an average of 10 tetrasaccharide repeats per polymer was generated using polycondensation reactions (25). Bacterial CPs were obtained from the laboratory strain of *S. pneumoniae* type 14 #883 isolated on April 22, 2012, from a child with acute otitis media at the Microbiology department of the “Scientific Center of Children’s Health” of the Ministry of Health of the Russian Federation (Moscow). The strain was expanded in semi-synthetic nutrient media. Isolation of CP was previously described (26). The presence of CP in the preparation was confirmed by NMR spectrometry.

### Animals

BALB/c male mice aged 6–8 weeks and 2 “Chinchilla” rabbits weighing 2.5 kg were purchased from the Scientific and Production Center for Biomedical Technologies (Moscow, Russia) and kept in the vivarium of the Mechnikov Research Institute for Vaccines and Sera. The housing, husbandry, blood sampling, and sacrificing conditions conformed to the European Union guidelines for the care and use of laboratory animals. Experimental designs were approved by the Mechnikov Research Institute for Vaccines and Sera Ethics Committee.

### Immunization

Mice were immunized intraperitoneally with tetra-BSA, hexa-BSA, and octa-BSA conjugates adjuvanted with aluminum hydroxide (Sigma-Aldrich Co., USA). Animals were dosed twice, on days 0 and 14 of the experiments. A single dose of glycoconjugates ranged from 10 to 1.25 µg (carbohydrate content) in twofold dilutions in saline. Aluminum hydroxide was added in an amount of 25 µL (250 µg) per immunizing dose and stored overnight at 4°C. Similar immunization schedules were used for immunization of mice with doses of the Prevenar-13 (Pfizer, USA) pneumococcal conjugate vaccine containing aluminum phosphate as an adjuvant, and doses of the Pneumo-23 (Aventis Pasteur, France) polysaccharide vaccine. Prevenar-13 murine vaccinations were single doses of either 2.2 or 1.1 µg per immunization (equivalent to 1 or 1/2 the recommended dose for humans). The Prevnar-13 vaccine contained *S. pneumoniae* type 14 CP conjugated with the inactive diphtheria toxin, CRM_197_, as the protein carrier. Pneumo-23 murine vaccinations were a single dose of 5 µg CP (corresponding to 1/5 of the dose recommended for humans). Antibacterial sera were recovered by repeated immunization of rabbits with inactivated *S. pneumoniae* type 14 bacteria.

### Measurement of Ab Response to CP and OS

Murine sera were collected 14 days after the second immunization with the OS conjugates, or with CRM_197_-CP included in the Prevenar-13 vaccine, as described earlier. Ab titers were measured by enzyme-linked immunosorbent assay (ELISA) from individual blood samples of 6–12 mice/dose (the results of two experiments), or from pooled sera of 6 mice/conjugate, as previously described (27). To prepare for the titer assays, flat-bottom plates (Biomedicals, Russia) were coated with *S. pneumoniae* type 14 bacterial CP (1 µg/well) or synthetic CP (0.5 µg/well). Wells were also coated with CP, tetra-BSA, hexa-BSA, and octa-BSA conjugates (0.4 µg/well) to determine murine post-immunization Ab titers. Rabbit anti-mouse peroxidase-conjugated IgG1 Abs (gamma 1 chain; Rockland Immunochemicals, Inc., USA) were used as secondary Abs.

Antibody titers to OSs of glycoconjugate-immunized mice were detected on streptavidin-coated 96-well plates (Pierce^®^, Thermo Scientific Inc., USA) with binding capacities of 5 pmol biotin/well. OS–biotin conjugates were adsorbed on streptavidin-coated 96-well plates at 15 pmol/well. Washes were performed with phosphate-buffered saline (PBS) (Sigma, USA) supplemented with 0.05% Tween 20 (Panreac Syntesis, Spain). ELISA was performed according to the manufacturer’s instructions. Briefly, 150 nM solutions diluted in PBS of each biotinylated OS (100 µL/well) were transferred into streptavidin-coated wells. OSs were incubated for 2 h with shaking (300 RPM) at room temperature. Each well was washed three times with 200 µL of wash buffer. Serial dilutions of antisera were prepared and added to each well. Plates were incubated for 30 min at room temperature. Each well was washed three times with 200 µL of wash buffer. Secondary rabbit anti-mouse peroxidase-conjugated IgG1 Abs or goat anti-rabbit peroxidase-conjugated IgG Abs (Thermo Scientific, USA) were added to each well. After 30 min of incubation with shaking (300 RPM) at room temperature, wells were washed three times with 200 µL of wash buffer. Enzyme substrate aliquots were added, followed by incubation for 15 min at room temperature. Optical densities (ODs) were determined with a microplate reader (iMark, Japan) at 450 nm. Antibody titers are expressed as dilution of serum or as log_10_ values.

### Antigen-Binding Capacity of Glycoconjugates-Induced Ab

To study the Ab-binding capacity in the sera of mice immunized with OS-conjugates, CP, or bacteria, biotinylated OSs were adsorbed on the bottom of streptavidin-coated 96-well plates (Thermo Scientific, USA) with a binding capacity of 5 pmol biotin/well. After adding immune antisera (90 µL/well), a concentration gradient of the OS ligands and synthetic CP or bacterial CP in PBS (1–10 µg/well) was inoculated into the wells. PBS (10 µL) was added in the control wells. The ELISA reaction procedures were the same as described earlier. Incubations with ligands and CP were carried out for 1 h at 37°C. Plates were washed three times with 200 µL/well of PBS-Tween 20. Next, working dilutions of peroxidase-conjugated rabbit anti-mouse IgG1 Abs (Rockland Immunochemicals, USA) or goat anti-rabbit peroxidase-conjugated IgG Abs (Thermo Scientific, USA) were added, as appropriate. Plates were incubated for 45 min at 37°C and then washed three times with 200 µL/well of PBS-Tween 20. Next, 100 µL of TMB was added per well to stain the bound reaction products. After 15 min, the reactions were quenched with 1 M H_2_SO_4_. ODs were determined at 450 nm with a microplate reader. The results were presented here as 50% inhibitory concentration (IC_50_) values. IC_50_ values were the concentrations of inhibitors that led to a twofold decrease of the OD and were calculated using calibration curves.

Flat-bottom plates (Biomedicals, Russia) coated with synthetic CP (0.5 µg/well) were also used. The procedure was the same as described earlier.

### Opsonophagocytosis Assay

The opsonizing activities of Abs elicited against the glycoconjugates were quantified by measuring uptake of heat-killed *S. pneumoniae* type 14 bacterial cells by neutrophils and monocytes in the peripheral blood of untreated mice. Heat-killed bacteria were labeled by fluorescein isothiocyanate (FITC). FITC-labeled bacteria (10^9^ cells/mL) were treated with sera (10 µL) from non-immunized mice (*n* = 6) or the antisera (10 µL) from mice (*n* = 6 for each conjugate) vaccinated twice with each of the synthetic conjugates (10 µg of carbohydrate) and then harvested 2 weeks after the second immunization. Either (1) heat-killed FITC-labeled bacteria, (2) heat-killed FITC-labeled bacteria treated by non-immune sera, or (3) heat-killed FITC-labeled bacteria treated with antiserum to each BSA conjugate were added to peripheral blood from non-immunized mice (*n* = 10). Sera and antisera were added to the bacteria at a 1:1 ratio and then incubated for 20 min at 37°C and washed by centrifugation (2,500 *g*, 10 min) in RPMI-1640 (Sigma, USA). The number of neutrophils and monocytes that internalized FITC-labeled bacteria was determined by flow cytometry (Cytomix FC-500, Beckman Coulter, USA, with CXP software). Cell population gates were determined by front and side light scattering and cell size; there were 10,000 cells/gate. The results were presented as the percentage of neutrophils or monocytes that phagocytized heat-killed FITC-labeled *S. pneumoniae* type 14 cells.

### Glycoconjugates-Induced Passive Protection in Naive Mice

BALB/c mice were intraperitoneally immunized once with 25 µL of pooled immune sera obtained from the glycoconjugate-exposed mice (*n* = 6) as described earlier; each single dose of each glycoconjugate was 10 µg (carbohydrate content) per mouse. Each serum was tested in eight unexposed mice. Control injections (25 µL) included sera from non-immunized mice given to unexposed mice (*n* = 8), and eight unexposed mice received only saline. Two hours after the serum inoculations, mice were challenged with intraperitoneal exposure to *S. pneumoniae* type 14. Mortality rates were calculated 7 days post-infection.

### Glycoconjugate-Induced Active Protection in Immunized Mice

BALB/c mice were immunized twice intraperitoneally with each glycoconjugate in twofold dilutions from 10 to 1.25 µg (carbohydrate content) per mouse on days 0 and 14. The same animals were challenged after 2 weeks interval with 5 × 10^8^ colony forming units of *S. pneumoniae* type 14/0.5 mL. Non-immunized control mice (8–10 animals/group) were also challenged with the type 14 bacteria. As reference agents, Pneumo-23 and Pevenar-13 were given at single doses of 5 and 1.1 µg of CP *S. pneumoniae* type 14, respectively, and injected according to the same schedule as described earlier. The results of three experiments were measured. Mortality rates were calculated at 7 days post-infection.

### Statistical Analysis

Groups were compared using Mann–Whitney rank sum tests for independent samples. Yates-corrected Chi-square tests were used to evaluate survival of mice after the challenges. *P* values of ≤0.05 were considered statistically significant (STATISTICA 10 software).

## Results

### Anti-CP Response Induced by the Glycoconjugates

**Figure 2:**
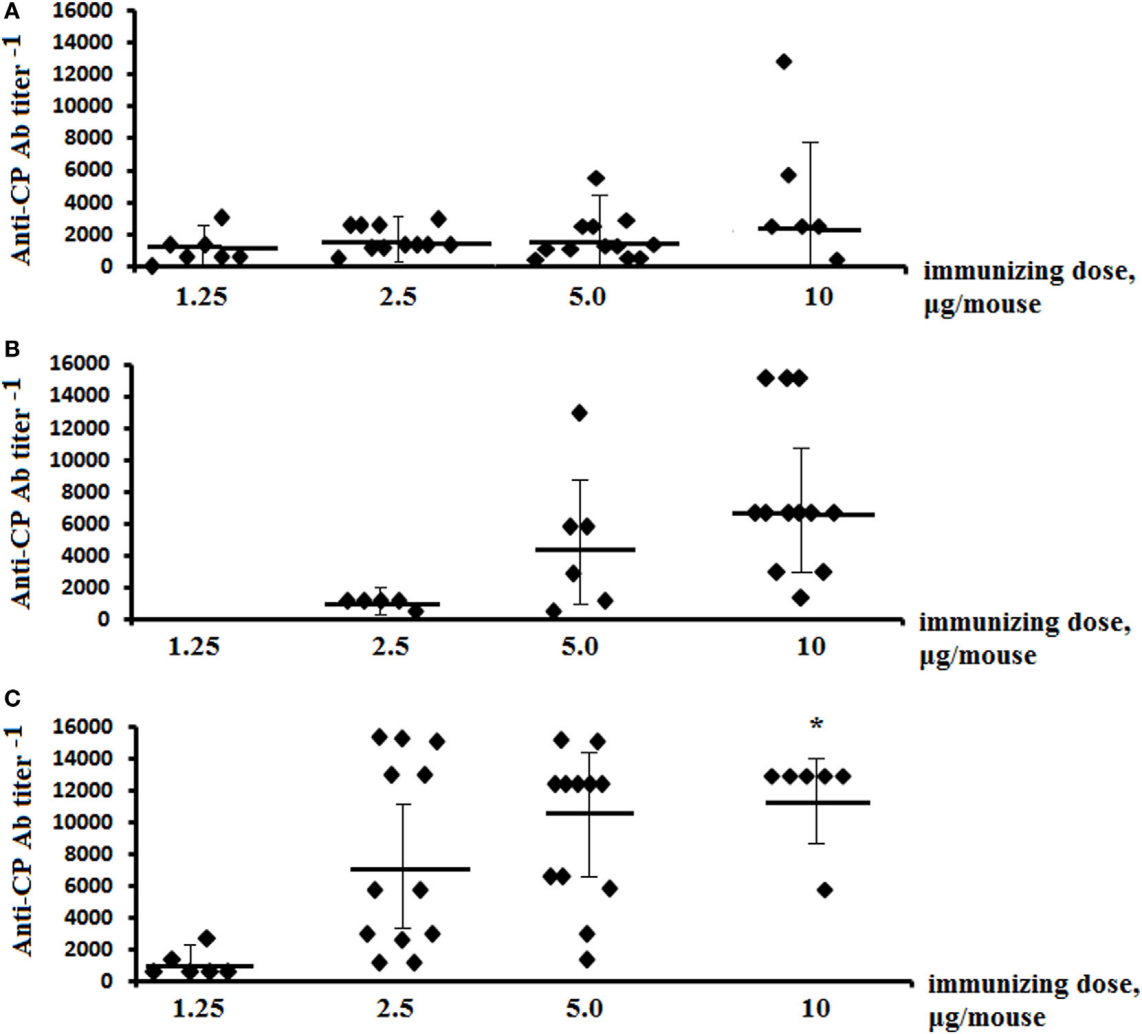
Anti-CP IgG1 antibody (Ab) titers in mice immunized with the glycoconjugates. BALB/c mice were immunized intraperitoneally with tetra-bovine serum albumin (BSA) **(A)**, hexa-BSA **(B)**, and octa-BSA **(C)** conjugates adsorbed on aluminum hydroxide twice over 14 days at 1.25–10 µg/dose (the hexa-BSA conjugate was injected at 2.5–10 µg/dose). Anti-CP IgG1 Ab titers in murine blood sera were determined by enzyme-linked immunosorbent assay 2 weeks after the second immunization. *Streptococcus pneumoniae* type 14 bacterial CP was used as the coating antigens. The data from two experiments were summarized. For each glycoconjugate, blood was taken from 6 to 12 mice. The data represent individual anti-CP IgG1 Ab titers, bars indicates median ± SD. Mann–Whitney Rank Sum tests were used to evaluate significance. Differences in the anti-CP IgG1 Ab titers between tetra-BSA and octa-BSA conjugates at the immunizing dose of 10 µg/mouse, *P < 0.05.

Only IgG1 Ab titer levels were assessed in the sera of glycoconjugate-immunized mice, as the level of IgG2a, IgG2b, and IgG3 was previously shown to be low in exposed animals (26). Post-immunization anti-CP Ab titer levels were determined in individual murine blood sera in ELISA using *S. pneumoniae* type 14 CP as the coating antigen (Figure 2). The highest IgG1 Ab titers were induced against glycoconjugate injections at the dose of 10 µg (carbohydrate content). Ab titers to tetrasaccharide conjugates (Figure 2A) had no difference with hexasaccharide conjugates (Figure 2B) and were lower than to the octasaccharide conjugates (Figure 2C) (*P* < 0.05).

**Figure 3:**
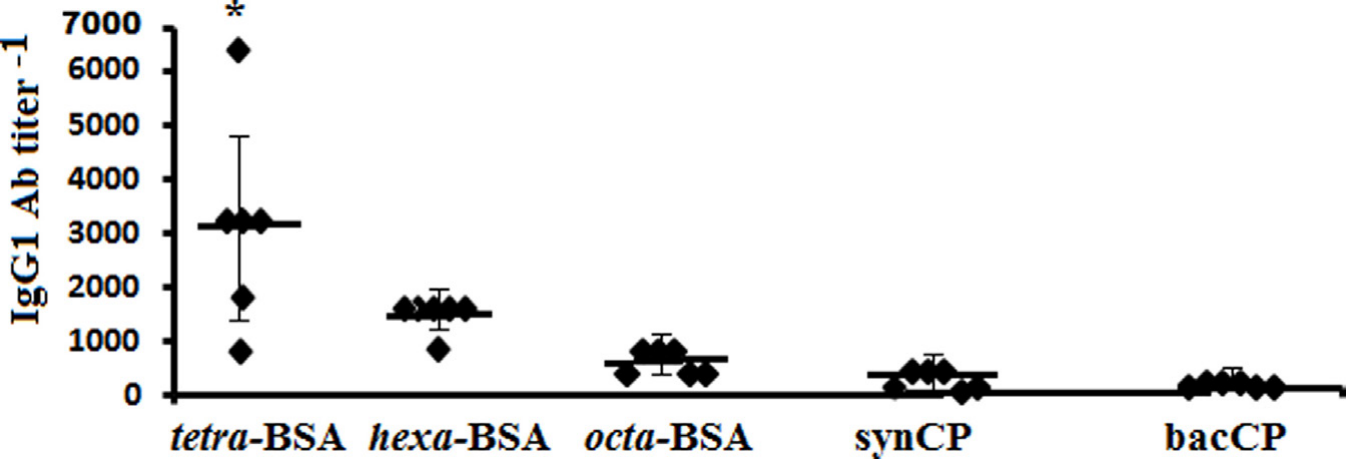
IgG1 antibody (Ab) titer in mice immunized with *Streptococcus pneumoniae* type 14 CP. BALB/c mice received two intraperitoneal immunizations with the conjugated pneumococcal vaccine Prevenar-13 with aluminum phosphate as adjuvant at 1.1 µg (content of *S. pneumoniae* type 14 CP) per dose. The tetra-bovine serum albumin (BSA), hexa-BSA, and octa-BSA conjugates, and synthetic (synCP) and bacterial CP (bacCP) were capture antigens, coating the enzyme-linked immunosorbent assay plates. The data are represented by individual titers of IgG1 Abs, bars indicate median ± SD. Mann–Whitney Rank Sum tests were used to determine significance. For differences in the level of Abs detected against tetra-BSA and octa-BSA conjugates, *P < 0.05.

IgG1 Ab titers in sera of mice immunized with conjugated pneumococcal vaccine Prevenar-13 (contains *S. pneumoniae* type 14 CP-CRM_197_) were evaluated using each neoglycoconjugates, synCP, and bacCP as coating antigens (Figure 3). The highest titers of IgG1 Abs in the same sera to Prevenar-13 were detected against the tetra-BSA conjugate, lower titers were detected against the hexa-BSA and octa-BSA conjugates, and the lowest titer of IgG1 Abs was detected against synCP and bacCP.

In contrast to *S. pneumoniae* type 14 CP, the tetra-BSA conjugate possessed the highest ability to recognize CP-induced IgG1 Abs.

### Anti-OS Response Induced by the Glycoconjugates

**Figure 4:**
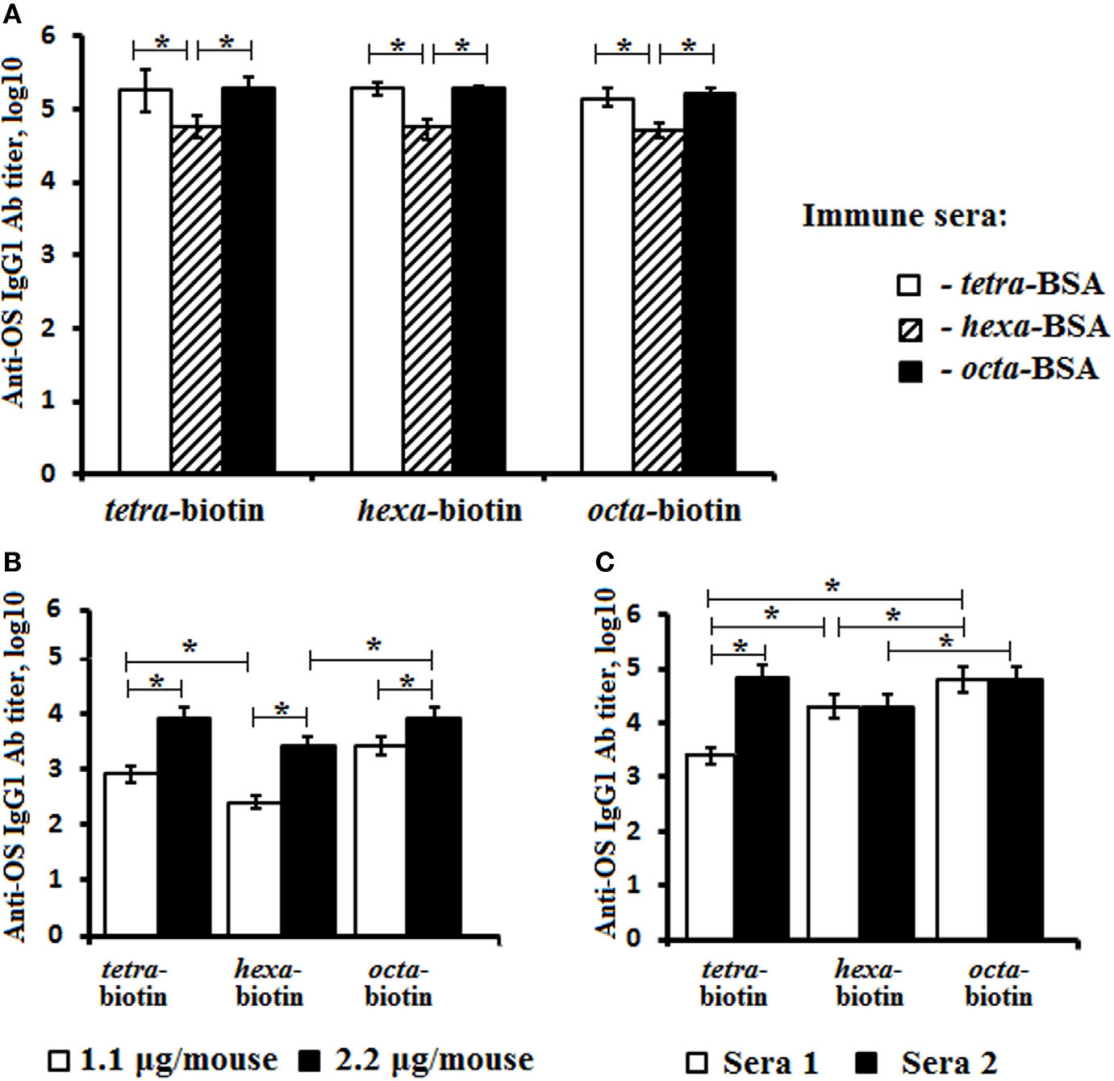
Anti-OS IgG1 antibody (Ab) titer in mice immunized with the OS-conjugates, CP, or bacteria. The biotinylated oligosaccharides tetra-biotin, hexa-biotin, and octa-biotin were applied as coating antigens. **(A)** Fourteen days after the second immunization, IgG1 Ab titers were determined in the pooled sera of BALB/c mice (*n* = 6) intraperitoneally vaccinated with a single dose (10 µg) of each conjugate adsorbed on aluminum hydroxide. **(B)** Fourteen days after the second immunization, the IgG1 Ab titers were measured in the pooled sera of two groups of BALB/c mice (*n* = 6) who were intraperitoneally vaccinated with single doses of either 1.1 or 2.2 µg (CP content) of *Streptococcus pneumoniae* type 14 CP-CPM_197_ adsorbed on aluminum phosphate. **(C)** The level of IgG Abs in rabbits (*n* = 2) immunized with inactive *S. pneumoniae* type 14 bacterial cells. Ab titers were transformed to log_10_. *n* = 6 assays per antiserum. The data are displayed as a mean value ± SD. Mann–Whitney Rank Sum tests were used to determine significance, *P < 0.05.

The titers of anti-OS IgG1 Abs were measured against biotinylated OSs in the pooled sera of glyoconjugate-immunized mice (Figure 4A). The highest levels of IgG1 anti-OS Abs were observed in the sera of mice immunized with the tetra-BSA and octa-BSA conjugates. The lowest level of Abs was detected in the serum generated by exposure to the hexa-BSA conjugate (*P* < 0.05).

Titer levels of IgG1 Abs to tetra-, hexa-, and octasaccharide in pooled sera from these two groups of mice immunized with the pneumococcal conjugate vaccine Prevenar-13 at doses of either 1.1 or 2.2 µg (CP content) were also measured using biotinylated OS (Figure 4B). Ab titers were higher with administration of the 2.2-µg/mouse dose, when compared with the lower dose (1.1-µg/mouse; *P* < 0.05). The titers of IgG1 Abs to the tetra- and octasaccharide in both pooled sera exceeded the level of Abs generated against the hexasaccharide (*P* < 0.05).

Investigations of two antibacterial sera obtained by repeated immunization of rabbits with inactivated bacterial cells revealed differences between the levels of anti-OS Abs detected using biotinylated OS (Figure 4C). In antibacterial serum 1, IgG Ab titers to the tetrasaccharide were lower than hexa- and octasaccharide Ab titers (Log_10_, 3.4 vs. 4.3 and 4.8, respectively; *P* < 0.05). Conversely, serum 2 had higher levels of IgG Abs against the tetra- and octasaccharides with the lowest level of Abs generated against the hexasaccharide (*P* < 0.05).

Antibodies in glycoconjugate-induced sera, as well as anti-CP and anti-pneumococcal whole cell sera of animals recognized synthetic OSs of different length.

### Antigen-Binding Capacity of the Glycoconjugate-Induced Abs

**Figure 5:**
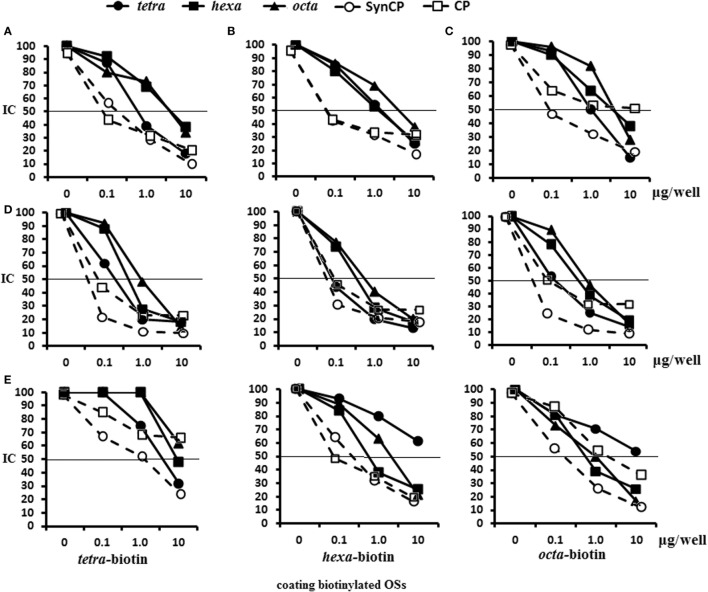
Inhibition of IgG1 antibodies (Abs) in mice immune sera with OS ligands and CP. Enzyme-linked immunosorbent assay inhibition assays were performed using streptavidin-coated plates with tetra-biotin, hexa-biotin, and octa-biotin adsorbed on their surfaces. **(A–C)** Inhibition of IgG1 Abs in the pooled sera of BALB/c mice (*n* = 6) that were immunized twice intraperitoneally with glycoconjugates at 10 µg/dose. Sera were obtained 14 days after the second immunization. Serum samples for all glycoconjugates were diluted 1:4,000. The tetra-, hexa-, and octasaccharide ligands, synCP, and bacCP were used as inhibitors and applied in amounts ranging from 0 to 10 µg/well. The horizontal line indicates the IC_50_ at the point of intersection of the inhibition curves. **(A)** Tetra-bovine serum albumin (BSA) conjugate antiserum was tested against tetra-biotin capture material. **(B)** Hexa-BSA conjugate antiserum was tested against hexa-biotin capture material **(C)**. Octa-BSA conjugate antiserum was tested against octa-biotin capture material. **(D)** Inhibition of IgG1 Abs was measured in the pooled sera of BALB/c mice immunized twice intraperitoneally over 2 weeks with the conjugated pneumococcal vaccine, Prevenar-13, at 1.1 µg of *Streptococcus pneumoniae* type 14 CP per single dose. The dilutions of sera tested against the tetra-biotin and octa-biotin coating antigens were 1:500; hexa-biotin was diluted 1:300. **(E)** Inhibition of IgG Abs was measured in serum harvested from rabbits that were immunized multiple times with inactivated *S. pneumonia* type 14 bacteria. The dilution of rabbit sera tested against tetra-biotin coating antigens was 1:300, against hexa-biotin and octa-biotin coating antigens was 1:3,000; *n* = 3 per data point.

Antigen-binding capacities of the Abs in the immune sera to OS-conjugates, CP, or bacteria were tested in ELISA using streptavidin plates coated with biotinylated tetra-, hexa-, and octasaccharides (Figure 5). Inhibition of binding was carried out by blocking binding reactions with biotinylated OS after incubation with the tetra-, hexa-, and octasaccharide ligands, as well as synCP and bacCP into immune sera.

In the system tetra-BSA antisera/tetra-biotin, the tetrasaccharide ligand had the highest inhibitory capacity, followed by synCP and bacCP (Figure 5A). For the hexa-BSA antisera/hexa-biotin, the best inhibitory activities were demonstrated by the tetra- and hexasaccharide ligands, synCP, and bacCP (Figure 5B). For the octa-BSA antisera/octa-biotin, the highest inhibitory activity possessed the tetrasaccharide ligand and synCP (Figure 5C). Taken together, the tetrasaccharide ligand had the maximal abilities to inhibit binding between anti-OS IgG1Abs and bound biotinylated OS.

In CRM_197_-CP-exposed sera/tetra-biotin, the tetrasaccharide ligand, as well as synCP and bacCP, had high inhibitory activity (Figure 5D). The tetrasaccharide ligand, as well as synCP and bacCP, inhibited interactions of anti-CP Abs with hexa-biotin in a higher degree than the hexa- and octasaccharide. The same results were obtained in the system CRM_197_-CP-antisera/octa-biotin. Thus, the tetrasacchride ligand exhibited maximal binding inhibitions between anti-CP IgG1 Abs and biotinylated OS.

For the first rabbit antibacterial sera (Sera 1), the tetrasaccharide ligand and synCP possessed the highest inhibitory activity against tetra-biotin (Figure 5E). Interestingly, when hexa-biotin was the coating antigen, high inhibitory activities were revealed for the hexa- and octasaccharide ligands, as well as for synCP and bacCP. Hexa- and octasaccharide, as well as synCP and bacCP, possessed high inhibitory activities against octa-biotin. In case of the rabbit antibacterial serum, the tetrasaccharide inhibited IgG Abs only in the tetra-biotin system, because when being tested against the hexa-biotin and octa-biotin, the working dilution of the serum was 1:3,000, and Abs to the tetrasaccharide fragment of CP (titer 1:3,200) could not be identified.

**Figure 6:**
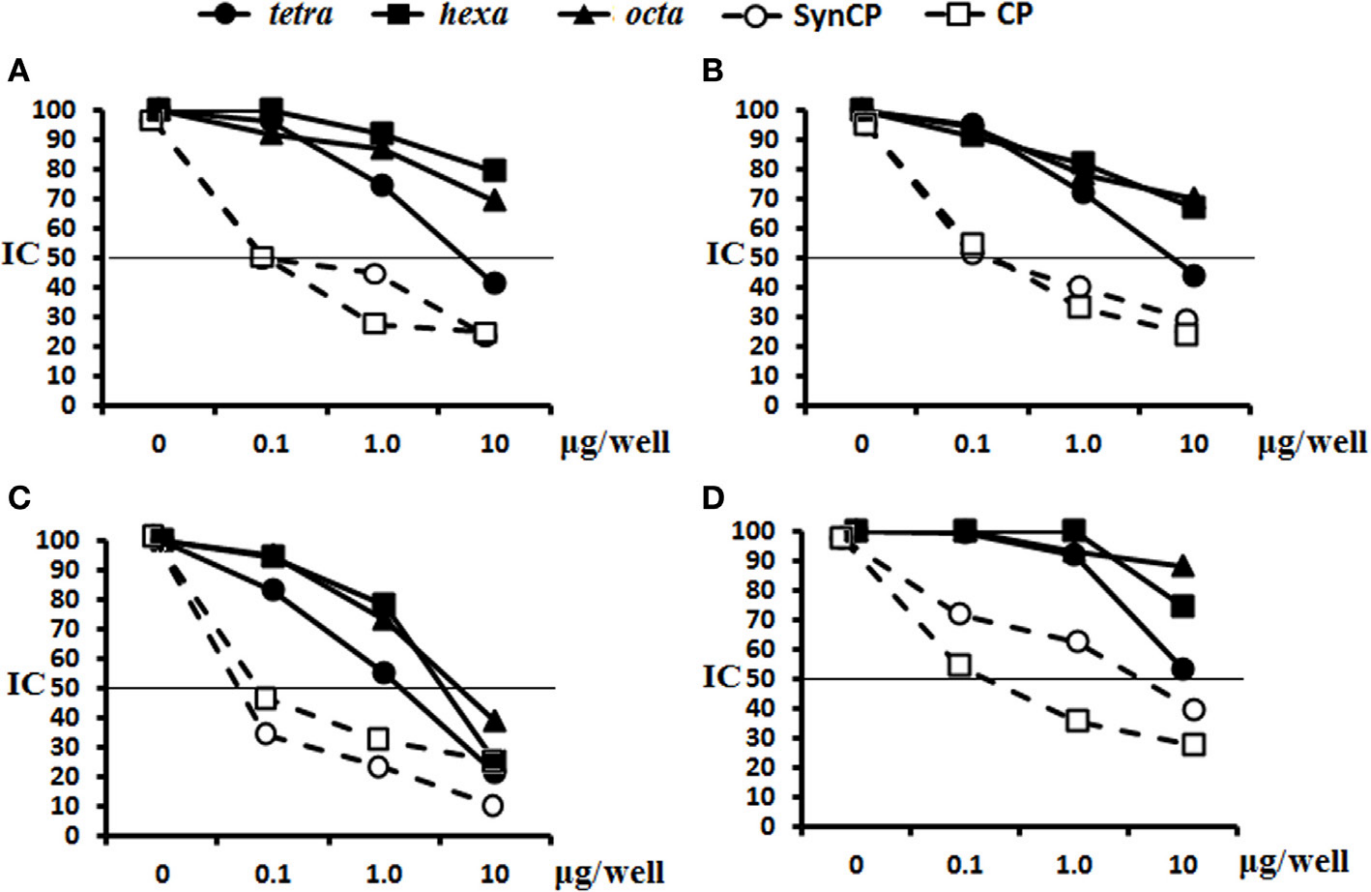
Inhibition of IgG antibodies (Abs) recognizing SynCP as the coating antigen with OS ligands and CP. Pooled immune sera were obtained after double intraperitoneal immunization of BALB/c mice (*n* = 6 for each glycoconjugate) with the OS-bovine serum albumin (BSA) conjugates adsorbed on aluminum hydroxide (10 µg of carbohydrate/single dose). The tetra-, hexa-, and octasaccharide ligands, as well as synCP and bacCP, were used as inhibitory materials at concentrations of 0–10 µg/well. The horizontal line indicates the IC_50_ level at the point of intersection of the inhibition curves. **(A)** Inhibition of IgG Abs was measured in tetra-BSA conjugate sera at a dilution of 1:400. **(B)** Inhibition of IgG Abs was measured in hexa-BSA conjugate sera at a dilution of 1:400. **(C)** Inhibition of IgG Abs was measured in octa-BSA conjugate sera at a dilution of 1:1,600. **(D)** Inhibition of IgG Abs was measured in *Streptococcus pneumoniae* type 14 CP-CRM_197_ at a dilution of 1:200. Immune sera were obtained after immunization of mice (*n* = 6) with Prevenar-13 adsorbed on aluminum phosphate at 2.2 µg (content of *S. pneumoniae* type 14 CP) per single dose.

The abilities of the OS ligands, as well as synCP and bacCP, to inhibit the binding of either glycoconjugate or CP antisera to synCP coating antigens were studied (Figure 6). In this case, IgG Abs were determined, as the level of IgG1 was universally low. For the sera of mice immunized with the tetra-BSA (Figure 6A), hexa-BSA (Figure 6B), and octa-BSA conjugates (Figure 6C), the tetrasaccharide ligand, as well as synCP and bacCP, possessed the highest inhibitory activities. The hexa- and octasaccharides only demonstrated inhibitory activities in the octa-BSA conjugate/synCP reactions. In the serum of mice immunized with *S. pneumoniae* type 14 CP*-*CRM_197_ (Prevenar-13), the highest inhibitory activities were detected for the tetrasaccharide ligand, synCP, and bacCP (Figure 6D). The tetrasaccharide ligand was the only one to reach IC_50_ for inhibiting interactions in the sera to Prevenar-13.

In general, these data provided clear evidence that the tetrasaccharide ligand possessed the best capacity to bind to anti-OS, anti-CP, and antibacterial Abs.

### Opsonophagocytic Capacity of Glycoconjugate-Induced Sera

**Figure 7:**
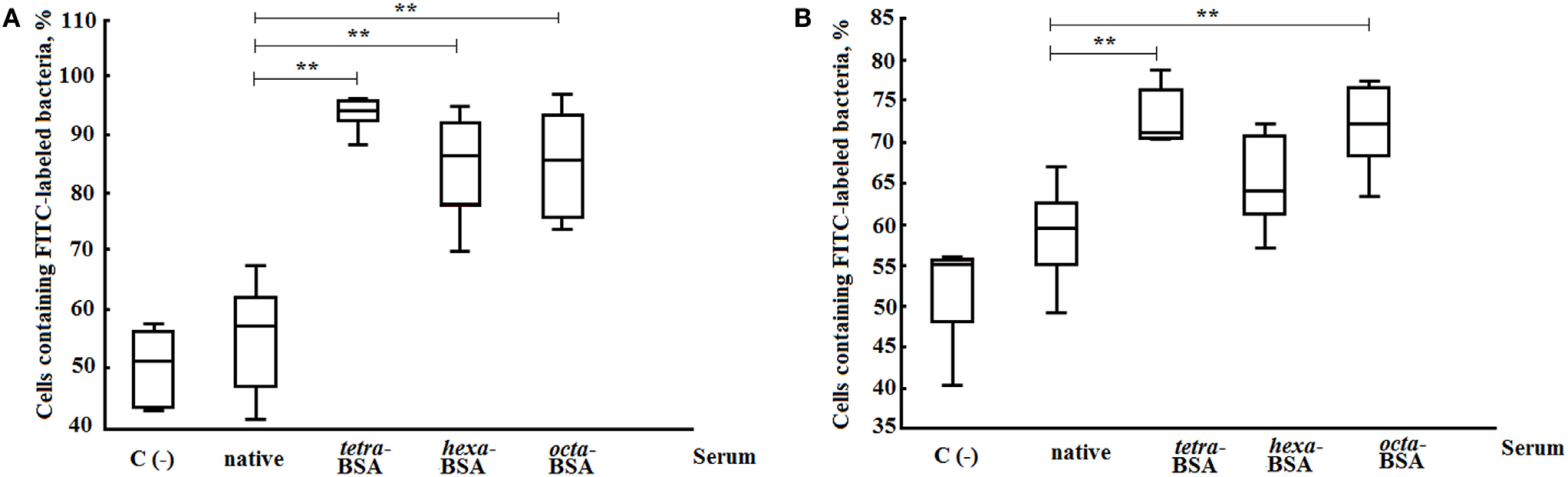
Opsonophagocytic capacity of the different elicited sera. **(A)** Neutrophils. **(B)** Monocytes. The following supplements were added to pooled peripheral blood samples from non-immunized BALB/c mice (*n* = 10): heat-inactivated FITC-labeled *Streptococcus pneumoniae* type 14 (C−), negative control; FITC-labeled bacteria with native serum; FITC-labeled bacteria treated with antisera obtained by immunization of mice with the tetra-bovine serum albumin (BSA), hexa-BSA, and octa-BSA conjugates adsorbed on aluminum hydroxide. Each antiserum was collected 14 days after immunization from mice (*n* = 6) that had been vaccinated with each of the glycoconjugates (10 µg/dose). Native serum was also obtained from non-immunized mice (*n* = 6). The numbers of neutrophils and monocytes that phagocytosed FITC-labeled bacterial cells were measured by flow cytometry. Box and Whisker plots represent the distribution of cells containing FITC-labeled *S. pneumoniae* type 14 bacteria. The box represents the 25–75% distribution; the enclosed line is the median, the Whiskers indicating the range from 2.5 to 97.5%. Mann–Whitney Rank Sum tests were used to calculate significance, **P < 0.01.

Opsonophagocytosis rates of heat-killed *S. pneumoniae* type 14 bacteria by neutrophils and monocytes collected from murine peripheral blood samples were examined by flow cytometry (Figure 7). The percentages of neutrophils (Figure 7A) and monocytes (Figure 7B) that phagocytosed heat-inactivated FITC-labeled bacterial cells of *S. pneumoniae* type 14 were quantified from blood samples of naive mice. The total number of active neutrophils that phagocytosed *S. pneumoniae* type 14 bacteria significantly increased in samples exposed to glycoconjugate antisera as compared with control samples without sera (C−), or samples supplemented with native sera (*P* < 0.01). No differences in the opsonizing rates between the antisera were revealed in this experiment. Similarly, monocytes captured *S. pneumoniae* type 14 bacterial cells in the presence of sera to the tetra-BSA and octa-BSA conjugates as compared with the native serum or the control (C−; *P* < 0.01). The lowest phagocytic activities of monocytes were observed after incubating bacteria with hexa-BSA conjugate antisera; no significant difference was found in monocyte phagocytosis rates of hexa-BSA-treated and native serum-treated bacteria.

To summarize, the Abs induced by the tetra- and octasaccharide conjugates possessed the highest opsonophagocytic capacity.

### Passive Protection Elicited by the Glycoconjugate-Induced Abs

Naive mice received a single intraperitoneal administration of glycoconjugate-induced serum followed by challenge with a lethal dose of *S. pneumoniae* type 14 (Table 1). The reference group of mice received native sera. Two hours after treatment, all groups of mice were challenged with a lethal dose of *S. pneumoniae* type 14. The number of mice surviving on day 7 (D7) after the challenge was given (Table 1). No serum was injected into control mice. The sera to the tetra-BSA and hexa-BSA conjugates protected six out of eight mice, while all the mice in the control group died (*P* < 0.01). The sera to the octa-BSA conjugate protected five out of eight mice (*P* < 0.05). The native serum protected three out of eight mice. Yates-corrected Chi-square tests, between the control and other groups, *P < 0.05, *P < 0.01. The titers of Abs to CP in the glycoconjugate-induced sera were significantly higher in comparison with the mice given native sera (Mann–Whitney Rank Sum test, *P < 0.05).

**Table 1:** Passive protection of mice with immune sera to the glycoconjugates.

Immune sera to glycoconjugates	Anti-CP antibodies titer, log_10_	Survivors	
		D0	D7
Tetra-bovine serum albumin (BSA)	3.65 ± 0.61*	8	6**
Hexa-BSA	3.82 ± 0.58*	8	6**
Octa-BSA	4.07 ± 0.60*	8	5*
Native sera	2.17 ± 0.36	8	3
Control	<2.0	8	0

*P < 0.05, ** P < 0.01.

Thus, all glycoconjugate-induced sera possessed the preventive capacities.

### Active Protection upon Challenge of Glycoconjugate-Immunized Mice

The results of three experiments of protective activities of glycoconjugates challenged with a lethal dose of *S. pneumoniae* type 14 are given (Table 2). In the first experiment (Exp. 1, Table 2), the tetra-BSA conjugate protected mice from infections (more than 4 LD_50_) at doses of 2.5, 5.0, and 10 µg compared with controls (naive mice; *P* < 0.01, *P* < 0.001, and *P* < 0.001, respectively). For the hexa-BSA conjugate, the optimal immunizing dose was 10 µg (*P* < 0.01). The octa-BSA conjugate protected mice at immunizing doses of 5 and 10 µg (*P* < 0.001 and *P* < 0.01, respectively). Yates-corrected Chi-square test, **P < 0.01, ***P < 0.001.

**Table 2:** Protective activity of the glycoconjugates.

Experimental series	Immunogen	Single dose (carbohydrate content) per mouse, μg	Survivors	
			D0	D7
1	Tetra-bovine serum albumin (BSA)	2.5	8	8***
		5.0	8	8***
		10	8	7**
	Hexa-BSA	2.5	8	1
		5.0	8	2
		10	8	7**
	Octa-BSA	2.5	8	3
		5.0	8	8***
		10	8	6**
	Control	Saline	8	0
2	Tetra-BSA	1.25	10	10***
	Octa-BSA	1.25	10	7**
	CP-CRM_197_	1.1	4	4***
	Control	Saline	10	0
3	Hexa-BSA	10	10	10***
	CP	5.0	10	2
	Control	Saline	10	1

**P < 0.01, *** P < 0.001.

In the second experiment (Exp. 2, Table 2), mice were immunized with lower doses (1.25 µg) of the conjugates and challenged with 4 LD_50_ of *S. pneumoniae* type 14. Here, the hexa-BSA conjugate was not tested, because in the previous experiment it did not protect the animals from infection at the lowest doses. The highest protection was induced by the tetra-BSA conjugate (*P* < 0.001), whereas the octa-BSA conjugate applied at the same dose was less effective (*P* < 0.01). Additionally, the *S. pneumoniae* type 14 CP-CRM_197_ conjugate at a dose equivalent to the doses of the BSA conjugates protected all mice from infections.

In the third experiment (Exp. 3, Table 2), the protective activity of the hexa-BSA conjugate was analyzed at the most effective single immunizing dose (10 µg of carbohydrate per mouse) and compared with the *S. pneumoniae* type 14 CP (5 µg of carbohydrate per mouse) from a commercial polysaccharide vaccine. In this case, 1.9 LD_50_ was used as the infectious dose of *S. pneumoniae* type 14, lower than in the two previous experiments. The CP administered without an adjuvant did not protect the mice against the infection, whereas the hexa-BSA conjugate adsorbed on aluminum hydroxide at the most effective single immunizing dose (10 µg/mouse) protected all immunized mice.

Thus, it was revealed that all neoglycoconjugates adsorbed on aluminum hydroxide possessed protective activity. The protective activity of the tetra-BSA conjugate was significantly higher than that of the hexa-BSA and octa-BSA conjugates (Exp. 1, Table 2). Given the strong protective capabilities of the tetra-BSA conjugate, the tetrasaccharide ligand seems the best of these candidates for development of an *S. pneumoniae* type 14 vaccine.

## Discussion

Selection of an optimal OS ligand possessing sufficient protective activity is the major barrier faced by developers of neoglycoconjugate vaccines. Modern chemical methods allow efficient syntheses of OSs representing potential protective epitopes of bacterial polysaccharides suitable for vaccine development (28–32). This study presented comparative immunological data of three potential protective epitopes of the *S. pneumoniae* type 14 CP. The tetra-, hexa-, and octasaccharide OS units differed from one another structurally and consisted of 1, 1.5, and 2 repeating units related to the CP of *S. pneumoniae* type 14.

The chemical structure of the OSs is an important factor in determining the immunogenicity of epitopes (15). Among the structurally unique tetra-, penta-, and hexasaccharides related to the *S. pneumoniae* type 14 CP, there exists a range of capabilities to stimulate induction of anti-CP IgG Abs (21, 33). It has been demonstrated that octasaccharides possessing different chemical structures and, generally, OSs with higher chain lengths are immunologically active with capacity to bind anti-CP Abs. Conjugates of long-chain OSs induce specific Abs, which subsequently promote phagocytosis of *S. pneumoniae* type 14 bacterial cells (21, 33, 34). Investigation of a large group of OSs (from tri- to dodecasaccharides) possessing varying chemical structures and conjugated to CRM_197_ revealed that the tetrasaccharide conjugate elicited Ab production and promoted the phagocytosis of *S. pneumoniae* type 14 bacteria. However, these data were not confirmed by *in vivo* protective activity assays of the conjugated tetrasacchride in animal models (21).

All glycoconjugates synthesized and reported here displayed immunogenic properties and elicited anti-CP and anti-OS IgG1 Ab production. Notably, the tetra-BSA conjugate possessed the highest protective properties in murine *S. pneumoniae* type 14 infection challenge models as compared with hexa-BSA and octa-BSA conjugates, but induced lower titers of anti-CP IgG1 Abs. Abs to the short-length OSs may have less opportunity to recognize chemically treated CP, as treatment may hide antigenic determinants; antigenic determinants may be more accessible to Abs generated against OSs with longer carbohydrate chains. However, we showed in a previous study that tetra-BSA conjugate-induced Abs more actively agglutinated live *S. pneumoniae* type 14 cells than did hexa-BSA - and octa-BSA -induced Abs; their activity equaled that of commercial serotype-specific rabbit pneumococcal antisera (35).

Conjugated tetrasaccharide as coating antigen in ELISA possessed higher diagnostic capabilities than CP detecting the higher level of CP-induced IgG1 Abs than either the conjugated long-chain octasaccharide or high-molecular weight CP. Probably, anti-CP IgG1 Abs did not fit precisely to the conformational epitope of CP of *S. pneumoniae* type 14, or because inappropriate epitopes were exposed on the surface of immobilized CP. Thus, the anti-CP IgG1 Ab titers reported in sera to commercial polysaccharide-based pneumococcal vaccines may occur lower than real levels detected in the same sera against tetrasaccharide conjugate. Therefore, it is reasonable to use the tetrasaccharide conjugate as the coating antigen in ELISA to determine anti-CP IgG1 Ab titers.

Post-vaccination, anti-OS IgG1 Ab titers measured in murine blood sera with ELISA using biotinylated OSs were higher than those detected using bacterial or synthetic CPs. The presence of shared carbohydrate fragments in the different OS ligands may explain the observation that each OS could cross-inhibit binding of other glycoconjugate Abs as well as the antibacterial sera. As a result, these experiments did not identify the types and abundance of anti-OS Abs present in the sera of animals exposed to CP. However, the tetrasaccharide ligand possessed the highest binding capacity to Abs generated in OS-immunized murine sera, as well as anti-CP and anti-pneumococcal whole cell sera.

All glycoconjugate-induced antisera promoted phagocytosis of *S. pneumoniae* type 14 bacterial cells by neutrophils and monocytes in peripheral blood of non-immunized mice. All antisera also induced passive protection of naive mice from pneumococcal infections. Similar preventive efficacies of each anti-OS serum may have occurred because the highest effective doses of each glycoconjugate were used to generate the immune sera. Perhaps, differences in opsonophagocytic and preventive activity of sera could be detected if lower immunizing doses for obtaining glycoconjugate-induced sera would be used.

Here, it was revealed for the first time that tetra-BSA, hexa-BSA, and octa-BSA conjugates adsorbed on aluminum hydroxide protected mice against *S. pneumoniae* type 14 infections. The feasibility of using aluminum hydroxide as an adjuvant to stimulate high levels of Ab production against conjugated synthetic OS was demonstrated previously (26). The tetra-BSA conjugate had the highest protective activity that was comparable to those demonstrated by the commercially available conjugated pneumococcal vaccine, Prevenar-13.

Based on the results of these immunological studies, including *in vivo* protective activity assays in murine models, we conclude that the tetrasaccharide corresponding to one repeating unit of CP is an excellent candidate for the development of a neoglycoconjugate *S. pneumoniae* type 14 vaccine. Additionally, tetrasaccharide-based coating antigens can be applied to ELISA for reliable determination of anti-CP Ab titers.

## Conclusion

The neoglycoconjugate of the tetrasaccharide, which is composed of one repeating unit of *S. pneumoniae* type 14 CP, induced the formation of opsonizing Abs and possessed stronger protective activity in mice challenged with the lethal doses of pneumococcal bacteria as compared to the hexa- and octasaccharide conjugates. These data support application of the tetrasaccharide as a component for development of synthetic or semi-synthetic pneumococcal vaccines.

## Ethics Statement

BALB/c male mice aged 6–8 weeks and two “Chinchilla” rabbits weighing 2.5 kg were purchased from the Scientific and Production Centre for Biomedical Technologies, Branch “Andreevka” (Moscow, Russia) and kept in the vivarium of the Mechnikov Research Institute for Vaccines and Sera. The housing, husbandry, blood sampling, and sacrificing conditions conformed to the European Union guidelines for the care and use of laboratory animals. The design of experiments was approved by the Ethics Committee of Mechnikov Research Institute for Vaccines and Sera.

## Author Contributions

EK planned the studies on Ab responses in mice and rabbits and summarized the results. NA studied the protective and preventive activities of sera in mice, opsonophagocytosis assays, and statistical analyses of data. EA performed the ELISA inhibition tests using different coating antigens and immune sera. NE summarized the results, compared the data with contemporary literature, and statistically analyzed the results. NY obtained the capsular polysaccharides and rabbit immune sera. ES performed the chemical syntheses of the oligosaccharides and analyzed the results. DY performed the chemical syntheses, conjugated the OS with protein carriers and analyzed the results. YT performed the chemical synthesis, conjugated the OS with protein carriers and summarized the results. MG summarized the results, and compared the data with contemporary literature. NN planned the study, analyzed the results, and compared the data with contemporary literature.

## Conflict of Interest Statement

The authors declare that the research was conducted in the absence of any commercial or financial relationships that could be construed as potential conflicts of interest.

## Abbreviations

CP, capsular polysaccharide; BSA, bovine serum albumin; OS, oligosaccharide; Ab, antibody; MALDI-TOF, matrix laser desorption ionization time-of-flight; ELISA, enzyme-linked immunosorbent assay.
